# Experiences of Older Adults Living with Dementia Eligible for a Money Follows the Person Program

**DOI:** 10.1093/geroni/igaf122.726

**Published:** 2025-12-31

**Authors:** Julie Robison, Ellis Dillon, Chae Man Lee, Deborah Migneault, Kristin Baker, Richard Fortinsky, Lisa Barry

**Affiliations:** University of Connecticut, Farmington, Connecticut, United States; University of Connecticut, Farmington, Connecticut, United States; University of Connecticut, Farmington, Connecticut, United States; University of Connecticut, Farmington, Connecticut, United States; University of Connecticut, Farmington, Connecticut, United States; University of Connecticut, Farmington, Connecticut, United States; University of Connecticut, Farmington, Connecticut, United States

## Abstract

We sought to identify mechanisms contributing to disparities for older persons living with dementia (PLWD) in access, utilization, and quality in Connecticut’s Money Follows the Person (MFP) program. MFP enables Medicaid-insured nursing home (NH) residents to move back to the community with Home and Community-Based Services (HCBS). We constructed a retrospective cohort of Connecticut MFP-eligible Medicaid-insured individuals age 55+ who were long-term (90+ days) NH residents between 2018-2022 (N = 29,050) from population-level claims and assessment data combined with MFP administrative records. 60.3% of MFP-eligible residents had dementia (race/ethnicity composition: 80.8% White; 11.2% Black; 5.4% Hispanic; 2.6% other). Overall, 15.3% of MFP-eligible residents applied to MFP (access), 27.6% of MFP applicants moved to the community within 2 years (utilization), and 76.0% of movers sustained community living for 60+ days (quality). PLWD had significantly lower rates of applying to MFP (9.4% vs 24.1%), moving (22.7% vs 30.6%), and sustaining community living (71.9% vs 77.8%). Black (28.8%) and Hispanic (30.6%) residents had significantly higher MFP application rates than White (12.3%) residents and similar likelihood of moving and sustaining community living. Despite Connecticut’s successful MFP program, rates of access, utilization and quality are significantly lower for PLWD, although disparities shrank at each step. PLWDs’ lower rates of MFP applications, moves, and sustained community living may indicate a disparity and/or selection effect as may differences by race/ethnicity. Growth in PLWD and variations by race/ethnicity necessitate identifying better solutions for community-living and improving concordance between design of MFP and HCBS programs and individual needs.

